# The Necessity for Higher Efficiency in Teaching and Practicing Dentistry

**Published:** 1912-02-15

**Authors:** C. N. Johnson

**Affiliations:** Chicago


### THE NECESSITY FOR HIGHER EFFICIENCY IN TEACHING AND PRACTICING DENTISTRY.

BY C. N. JOHNSON, M.A., L.D.S., D.D.S., CHICAGO.

To the practitioner of dentistry who is observant of the rapid strides made by the profession, both in its educational methods and in its daily practice, there is quite naturally a fair measure of pride in this achievement. This is perfectly legitimate and in so far as it serves as a stimulus to better things it is well, but the moment it begins to display itself in any spirit of self satisfaction or unctuous complacency, that moment it becomes dangerous. The most critical time in the history of a man or a nation is the moment of success, and the same may truly be said with reference to a profession. There is nothing more dangerous than a spirit of self adulation, and to be perfectly satisfied with one's self is to start on the downward road to deterioration.

It is well, therefore, that at times the weaknesses in our profession be pointed out, and in this view of the case we should welcome any just criticism which is meted out to us. It is true that we are sometimes subjected to critisism which is not only unjust and untrue, but which is positively harmful. Any stricture which is biased and wide of the mark does more harm than good, because its tendency is to tear down instead of build up, and it is always easier to say how a thing shall be done than to do the thing itself.

We have had men in our own ranks whose natural pessimism and inherited or acquired obliqueness of vision has led them to snarl at the profession on every possible occasion, but who have not in their own capacity done anything to build up the profession and place it upon a better basis. We have grown to tolerate these men, sometimes possibly to our detriment, because we have believed them to be either honest in their convictions or harmless in their results. Occasionally some of their strictures have had sufficient truth in them to accomplish a measure of good by stimulating us to better things, but usually their perverted ideas have led to little but irritation and disgust.

There are two evils which unjust criticism of our profession may bring about. It may lead to discouragement and bitterness in the profession and it may unduly prejudice the public against us. Our capacity for doing good is always limited by any suspicion or distrust of our intentions or abilities on the part of the public, and anything which erects a barrier between us and the people who need our services is harmful.

A prominent illustration of this is an article which appeared in the August, 1911, number of *Current Literature*, a magazine which has a wide circulation and which apparently lent its endorsement to the promulgation of ideas inimical to the reputation of American dentistry—or, in fact, of any dentistry. A very prejudiced and unwise article had been published in the London *Lancet* by Dr. William Hunter, pathologist to the London Charing Cross Hospital, attacking dentistry as working a most baneful influence on humankind, and it was this article which *Current Literature* so unjustly exploited.

Dr. Hunter's contention is that "gold fillings, gold caps, gold bridges, gold crowns, fixed dentures, built in, on, and around diseased teeth, form a veritable mausoleum of gold over a mass of sepsis to which there is no parallel in the whole realm of medicine or surgery." And then he says: "There is no rank of society free from the fatal effects on health of this surgical malpractice. I speak from experience. The worst cases of anemia, gastritis, colitis of all kinds and degrees, of obscure fever of unknown origin, or purpura, or nervous disturbances of all kinds ranging from mental depression up to actual lesions of the cord, of chronic rheumatic affections, of kidney disease, are those which owe their origin to, or are gravely complicated by, the oral sepsis produced in private patients by these gold traps of sepsis. Time and again I have traced the very first onset of the whole trouble of which they complained to a period of a month or two of their insertion. The sepsis hereby produced is particularly severe and hurtful in its effects. For it is dammed up in the bone and in the periosteum, and cannot be got rid of by any antiseptic measures which the patient or the doctor can carry out. Moreover, it is painless, and its septic effects therefore go on steadily accumulating in intensity without drawing attention to their seat of origin.

"Such are the fruits of this baneful so-called 'conservative dentistry.' The title would be a fitting one if the teeth were a series of ivory pegs planted in stone sockets. But the teeth being what they are—namely, highly developed pieces of bone tissue, possessing, I would point out, a richer blood and nerve supply than any piece of tissue of the same size in the whole body—and planted in sockets of bone with the closest vascular relations to the bone and the soft tissues of the periosteum and the gums, the title that would best describe the dentistry here referred to would be that of 'septic dentistry.' Conservative it is, but only in one sense. It conserves the sepsis which it produces by the gold work it places over and around the teeth, by the satisfaction which it gives the patient, by the pride which the dentist responsible for it feels in his 'high-class American' work, and by his inability or unwillingness to recognize the septic effects which it produces."

Dr. Hunter's entire article is along the same intemperate lines, and is necessarily very misleading to the public in giving the impression that all dentistry is harmful. From beginning to end he says not one word favorable to dental service of any kind, nor does he make any distinction between good dentistry carefully performed under proper hygienic measures, and the sort of dentistry which places fillings, crowns and bridges in and around "diseased teeth.' It does not seem to be within Dr. Hunter's range of observation that teeth may be treated for disease and rendered healthy before fillings or crowns are inserted, and that it is one of the tenets of our professional faith that this shall invariably be done. His repeated flings at American dentistry are most unfortunate but they must be allowed to pass quite unworthy the dignity of Americans to notice. Dr. Hunter never would have made them if he had been personally and professionally acquainted with the representative practitioners of dentistry in America.

But let us not look at this incident wholly in the spirit of resentment. Let us try to profit by it in the sense of becoming better men by reason of it. While it is true that a grave injustice has been done to American dentistry on the part of Dr. Hunter, and particularly in the fact that his ideas should have been exploited by an American magazine, yet we must not shut our eyes to the fact that there is too much dentistry being done along the improper and unscientific lines pointed out by Dr. Hunter, and that the better element in the profession must lose no opportunity of recording its disapproval of such practice. Our professional honor should be one of our most precious possessions, and this we can never maintain till the rank and file are stimulated in their conscience and elevated in their ability. No man should assume to call himself a dentist who is not fully alive to the factor of sepsis as it affects the welfare of the patient, and no dentist should ever apply a crown to the root of a tooth without first seeing to it that the root is in a normal condition. Not only this, but in applying the crown he should aim to get such adaptation and have the root so perfectly protected that subsequent sepsis is impossible.

These things have been taught in the profession ever since crown work was begun, and to say that dentists are ignorant or indifferent regarding the significance of sepsis is to misstate the facts. Some men are careless and some are worse. In every calling there is always the irresponsible element, and it is this element which we should strive to reform. The need today is for the higher minded ethics of the profession to be carried into the daily practice of every dentist. Not merely that a man shall talk and write glibly of the necessity for hygienic considerations in the performance of our work, but that he shall day after day in the privacy of his own office enter into the scientific spirit of his operations, and not content himself till each case has been subjected to the closest scrutiny to bring it under the requirements of the latest and most approved principles of aseptic and hygienic dentistry.

Never in the history of the profession has the call been more urgent than now for self sacrificing and painstaking effort on the part of the dentist. Never before has public attention been so focused upon him. Our educational campaigns all over the country are directing the attention of the people to what we are doing and to the significance of our work, and we are to be weighed in the balance in the eyes of the public. It were fatal to our future usefulness if we in any way fail to measure up to the expectations which today are being built as to our efficiency in conserving human comfort and prolonging human life, and when we know in our inmost hearts what can be done for humanity by the application of the most approved and scientific methods of dentistry in ministering to human needs, it is something akin to criminality for us to neglect any opportunity of keeping faith with the people. And if we keep faithfully and unreservedly, not one man or two or a hundred, but every man and woman who today is practicing dentistry, then shall there be no warrant or excuse or even the possibility of excuse for making such wholesale and apparently vindictive charges as Dr. Hunter has made against dentistry in the article quoted. But if a few persist in doing indifferent work just so long will isolated cases be picked out by our critics and the baneful effects of these cases will be heralded widecast to the discredit of the entire profession. In view of the case it does seem at this time particularly important for the conscience of the profession to be quickened to the highest pitch so that every bit of service which we perform for the people shall reflect credit upon our calling rather than bring it into disrepute.

I look upon our professional situation today with mingled sentiments of hope and anxiety. I never was so hopeful as now regarding the future possibilities of our calling, and I never was more keenly anxious lest we allow the golden moment of our opportunity to pass us by and leave us without the full fruition of our rightful heritage. The issue lies with us. If we do our duty as practitioners day in and day out, at the chair and the bench, if we approach our work in the spirit of a serious professional and humanitarian responsibility, and meet each situation as if the eyes of the world were upon us, then not all the powers that be, outside our ranks, can injure us in the estimation of the world. If we do our whole duty we can afford to ignore such baseless strictures as have been cast upon us in the article quoted—in fact, such strictures will never be printed because they will never be written.

The closing paragraph of this article probably carries with it greater injustice than anyhting else contained therein. It says:

"There seems only too good reason to infer, moreover, that the professional education of dentists is often of the most superficial sort. The profession is recruited in some American communities almost haphazard. The training is frequently of the manual kind. Provided the student displays a digital dexterity in manipulation, he is not questioned too closely sometimes regarding his scientific knowledge. He goes about his work in the densest ignorance of such things as therapeutics and infection. He is no scholar and in no sense a man of science."

It has not been my privilege to read Dr. Hunter's original article in the *Lancet*, but it is difficult to believe that he should have given utterance to so gross a libel on our educational institutions as is herein contained. Those of us who are actively engaged in educational work are among the last to claim that perfection has been reached in our methods or our systems, and yet we can say without arrogance and with a reasonable degree of modesty that so far as an adequate preparation for their life work is concerned dental students are better prepared by their courses in college than are students of almost any other calling. In fact, the statement has been made by an eminent practitioner of medicine who is also dean of an honored and reputable medical college that, as a general rule dental students are better equipped on the day of their graduation to go out and intelligently administer to the dental needs of the people than are medical students equipped to administer to the medical needs. To say that the dental student "goes about his work in the densest ignorance of such things as therapeutics and infection," is to slander the intelligence of a large body of young men who are today being graduated from our colleges. These things are taught as carefully and with as much emphasis of their importance, as they are in the best medical colleges, and if at times a graduate lapses from the principles taught him in college and does not live up to his highest possibilities the same thing may justly be charged against the medical graduate. There is no intent in all this of resorting to the "pot calling the kettle black" kind of argument, but it must be remembered that young men are pretty much alike in all walks of life and that a few black sheep are likely to enter any fold.

In most of our dental colleges in America the training embraces something more than the "manual kind." It includes anatomy, physiology, histology, bacteriology, pathology, chemistry, physical diagnosis, materia medica and therapeutics, as well as the other so-called "practical branches," and no man can justly say that the training is not thorough. These fundamental subjects are all amply demonstrated in the dissecting room and the various scientific laboratories with which every progressive college is well equipped.

If manual training is made a prominent feature of dental college instruction, it is simply because that kind of training is absolutely necessary to properly equip a young man to skillfully serve the people. If there is any one thing pathetic in dental circles it is to see the man who has laboriously stored his mind with all of the theories incident to a college course but who lacks the digital skill to properly apply those theories. The only man who is more unfortunate than he, is the one who has neither the theory nor the manual aptitude.

To say that dental colleges ignore the scientific in their instruction is to make a mis-statement of fact, as any unprejudiced investigator may learn by visiting our colleges and noting the character of the instruction. In view of the brief period in which dental colleges have been in existence the present degree of perfection to which they have brought their work is something of which the profession as a whole need not feel ashamed. And yet in the same breath, I must acknowledge that I do not like that word "perfection." Nothing in all the universe is perfect, least of all are educational methods, which in order to serve the best purpose must be subject to constant evolution. And it is in the spirit of seeking the most certain way of evolving to better things that we should accept even so unjust an arraignment as that contained in *Current Literature*. Some of the most progressive men in our teaching ranks have pointed out in the recent past that there are certain subjects which may be more effectively taught in our colleges than they are, and every energy is being directed to accomplish this. The two subjects most often referred to are pathology and diagnosis, and in some schools these subjects are being emphasized today in a manner commensurate with their importance. It remains for all schools to recognize the fundamental bearing of these subjects on the future practice of the graduate, and to send him out better equipped than he has been in the past to meet the emergencies imposed on him by an ignorance of these fundamentals.

In making a plea for higher efficiency in teaching and practicing dentistry, I am impelled by no rancor toward any of our critics. I have a theory that we should all be helpful to one another whether in the capacity of a critic or a commendator, and so I am inclined to respect the opinions of my fellow man. But I have always been so intensely jealous of the fair name and reputation of my profession that I am keenly touched when any unjust reflection is cast upon it, and in view of the fact that I do not believe in retorting in kind there is only one course open to my mind to overcome the difficulty. This is by a general elevation of our efficiency as a profession to the extent of making criticism uncalled for or even impossible. When we have attained to that we need fear no shaft which may be directed against us, and in this connection it is well for us always to remember that it is more profitable to expend our energy in perfecting our methods than it is in explaining and defending our mistakes.—*Dental Review.*

